# Association of serum Interleukin-8 level with lymph node metastasis and tumor recurrence in gastric cancer

**DOI:** 10.3389/fonc.2022.975269

**Published:** 2022-09-16

**Authors:** Xiang Li, Guiping Xie, Jing Zhai, Yani He, Tongya Wang, Yaohui Wang, Lizong Shen

**Affiliations:** ^1^ Department of Surgical Oncology, Jiangsu Province Hospital of Chinese Medicine, Affiliated Hospital of Nanjing University of Chinese Medicine, Nanjing, China; ^2^ Department of Pathology, Jiangsu Province Hospital of Chinese Medicine, Affiliated Hospital of Nanjing University of Chinese Medicine, Nanjing, China

**Keywords:** gastric cancer, interleukin-8, lymph node metastasis, tumor recurrence, tumor marker

## Abstract

The level of pretherapeutic serum interleukin-8 (sIL-8) has been demonstrated to correlate with chemoresistance in gastric cancer. However, its clinicopathological significance of sIL-8 in gastric cancer remains unknown. Herein, a total of 335 patients diagnosed with gastric adenocarcinoma were enrolled. The clinicopathological features were collected, and the sIL-8 levels were measured using enzyme-linked immunosorbent assay. The sIL-8 levels ranged from 1.48 pg/ml to 1025.22 pg/ml with > 15.41 pg/ml defined as high according to the receiver operating characteristic analysis. sIL-8 levels were strongly associated with Lauren classification and tumor recurrence. High sIL-8 correlated with lymph node metastasis (LNM) in the intestinal- and diffuse-type tumors and acted as an independent risk factor for LNM in both types. Patients with high sIL-8 levels had worse relapse-free survival than those with low sIL-8 levels. High sIL-8 level was associated with tumor relapse in the intestinal- and diffuse-type tumors, and was also an independent risk factor in the intestinal- and mixed-type tumors. Further analysis revealed that sIL-8 levels were positively associated with LNM and tumor relapse in patients with negative carcinoembryonic antigen (CEA), but not in those with elevated serum CEA levels. In conclusion, this retrospective study demonstrated that the pretherapeutic sIL-8 level has predictive value for LNM and tumor recurrence, and may serve as a potential tumor marker in gastric cancer.

## Introduction

Gastric carcinoma remains the fifth most common malignancy and the third leading cause of cancer-related deaths worldwide, with a much higher incidence in Eastern Asia (1). Lymph node metastasis (LNM) is the prominent route of gastric cancer dissemination, and serves as the most important hallmark of tumor progression, and heralds the dismal prognosis in patients with gastric cancer (2). Albeit many progresses in early detection and comprehensive management of gastric cancer have been made in the past decades, the overall survival of gastric cancer patients remains low with approximately 40% to 60% recurrence rate after radical gastrectomy (3). Several classic tumor markers, such as carcinoembryonic antigen (CEA), have been currently used for screening or monitoring gastric cancer. However, they cannot meet the clinical demands due to low sensitivity and/or low specificity. It is imperative to identify more effective markers for gastric cancer metastasis and/or recurrence.

Previously, we demonstrated that the pretherapeutic serum interleukin-8 (sIL-8) levels correlate with chemoresistance to cisplatin in gastric cancer (4). However, the clinicopathological significance of sIL-8 in gastric cancer remains unknown. As a proinflammatory chemokine, IL-8 is mainly responsible for attracting neutrophils to injury and inflammation sites (5–7). Physiologically, monocytes, endothelial cells, and several epithelial cells can produce IL-8 (8, 9). In cancer, IL-8 has been revealed to be produced by tumor-associated macrophages (TAMs) (10). We have revealed that sIL-8 is mainly derived from cancer-associated fibroblasts (CAFs) in the gastric cancer (4). A growing number of studies have reported that IL-8 plays an important role in promoting tumor development. High sIL-8 levels are considered to be associated with larger tumor sizes, advanced stages, and poor prognoses in many cancer types, including breast cancer, prostate cancer, lung cancer, melanoma, colorectal cancer, and pancreatic cancer (11–13). Furthermore, several studies have suggested that sIL-8 can act as a negative prognostic biomarker in some solid tumors, although further validation is still lacking (8, 14).

CEA is the most commonly used biomarker of gastrointestinal malignant diseases in clinical practice. As a glycoprotein involved in cell adhesion, CEA is usually produced by gastrointestinal tissues and presents at a relatively high level during fetal development. Its level remains low in the blood of healthy adults (15, 16). However, serum CEA levels are significantly increased and are associated with poor prognosis in various types of adenocarcinoma, including colon, gastric or breast cancer (17–20). The prevalence of high serum CEA in gastric cancer ranges from 16% to 68% (21). Although serum CEA level is effective in monitoring gastric cancer patients with high CEA level, the convincing markers with effectiveness and convenience are still lacking in patients with normal serum CEA levels at the initial diagnosis. Furthermore, CEA may be occasionally detected as false-positive due to other non-malignant conditions or environmental exposures (15, 22).

The present study included 335 patients with gastric adenocarcinoma who had not undergone preoperative therapy. The clinicopathological features of these patients were collected and the pretherapeutic sIL-8 levels were assayed to investigate the clinical significance of sIL-8 in gastric cancer. sIL-8 level was revealed to be strongly associated with Lauren classification and tumor recurrence. Relapse-free survival (RFS) analyses indicated that high sIL-8 levels are associated with tumor recurrence in both intestinal- and diffuse-type tumors. High sIL-8 level is an independent risk factor for LNM or tumor recurrence in gastric cancer patients. Further analysis revealed that sIL-8 levels are positively associated with LNM and tumor recurrence in patients with negative CEA, but not in those with elevated serum CEA levels. Collectively, this retrospective study demonstrated that pretherapeutic sIL-8 level is associated with LNM and tumor recurrence, and may act as a potential tumor marker in gastric cancer, especially in patients with negative CEA.

## Materials and methods

### Patients and peripheral blood samples

A total of 335 patients diagnosed with primary gastric adenocarcinoma *as per* the American Joint Committee on Cancer (AJCC) criteria between February 2018 and December 2020 at the Department of Surgical Oncology, Affiliated Hospital of Nanjing University of Chinese Medicine, were enrolled in this study. All enrolled patients were admitted without obvious infectious diseases. All patients had not received preoperative chemotherapy or chemoradiotherapy, and underwent radical gastrectomy with curative intent. Peripheral blood samples were collected preoperatively following written consent according to an established protocol approved by the Institutional Review Board of Nanjing University of Chinese Medicine. The clinicopathological features were collected, including sex, age, depth of invasion (T stage), LNM (N stage), TNM stage, Lauren classification (intestinal, diffuse, or mixed type), tumor recurrence, and values of pretherapeutic serum tumor markers, including CEA, alpha-fetoprotein (AFP), carbohydrate antigen 199 (CA199), CA125 and CA153. All these pathological features were reviewed by two experienced pathologists. All these patients were followed up until December 2021 with an average follow-up duration of 731.66 days. This study also complied with the Declaration of Helsinki.

### Enzyme-linked immunosorbent assay

Human IL-8 enzyme-linked immunosorbent assay (ELISA) Kit (EH005-96, ExcellBio, China) was used to measure serum IL-8 levels of the enrolled patients. Assaying procedures were performed *as per* the manufacturer’s protocols. Each experiment was repeated at least three times.

### Receiver operating characteristic analysis for the threshold of sIL-8

Receiver operating characteristic (ROC) analysis was performed to evaluate the relevance of sIL-8 levels with tumor recurrence. The Youden index (sensitivity + specificity − 1) was calculated, and the sIL-8 level corresponding to the maximum Youden index was set as the threshold.

### Statistical analyses

RFS analyses were performed from the date of surgery till the detection of tumor recurrence. Kaplan–Meier curves were generated and compared using a log–rank test using GraphPad Prism software (version 8.0; La Jolla, CA). Pearson’s chi-squared test and Fisher’s exact test were used to compare the tumor characteristics and clinical data illustrated as cross-tables. Logistic regression analyses were used in univariate and multivariate analyses for LNM and tumor recurrence. All analyses were performed using Statistical Package for the Social Sciences software (version 25.0; IBM, Armonk, New York). A *P* value of < 0.05 was considered statistically significant.

## Results

### Clinicopathological features of enrolled patients

In the 335 enrolled patients, intestinal-, diffuse-, and mixed-type tumors accounted for 37.31% (125 cases), 19.40% (65 cases), and 43.28% (145 cases), respectively. **Table 1** demonstrated that older patients (aged > 60 years) constituted the majority in this cohort (68.66%); however, patients with diffuse-type tumor tended to be younger (*P* < 0.001). Males are still the main population of gastric cancer (71.34%), whereas female patients were significantly more likely to have diffuse-type disease (*P* < 0.001). With regard to patients with intestinal-type tumor, there were more cases of advanced T stage (*P* < 0.001) and N stage (*P* < 0.001) in patients with diffuse- or mixed-type diseases. Accordingly, more advanced TNM stage was detected in patients with diffuse- or mixed-type tumors (*P* < 0.001), with a much higher incidence of recurrence than patients with intestinal-type tumor (*P* = 0.008). We also showed that the traditional tumor markers, including CEA, AFP, CA199, CA125 and CA153, were not associated with Lauren classification (*P* > 0.05).

**Table 1:** Clinicopathological features of the enrolled 335 patients with gastric cancer.

Clinicopathological features	N	Lauren classification			*P*
		intestinal	diffuse	mixed	
Age (y)					< 0.001*
≤ 60	105	33	37	35	
> 60	230	92	28	110	
Sex					< 0.001*
male	239	104	31	104	
female	96	21	34	41	
Depth of invasion (T)					< 0.001*
T1	90	54	11	25	
T2–T4	245	71	54	120	
Lymph node metastasis					< 0.001*
N0	137	77	21	39	
N1–N3	198	48	44	106	
Distant metastasis (M)					N/A
M0	335	125	65	145	
M1	0	0	0	0	
TNM stage					< 0.001*
I	107	63	11	33	
II–IV	228	62	54	112	
Recurrence					0.008*
recurrence-free	262	109	46	107	
recurrence	73	16	19	38	
CEA					0.309
< 5 ng/ml	291	104	58	129	
≥ 5 ng/ml	44	21	7	16	
AFP					0.177
< 25 μg/l	327	124	64	139	
≥ 25 μg/l	8	1	1	6	
CA125					0.210
< 35 u/ml	324	119	65	140	
≥ 35 u/ml	11	6	0	5	
CA199					0.703
< 37 u/ml	301	114	59	128	
≥ 37 u/ml	34	11	6	17	
CA153					0.268
< 28 u/ml	333	125	65	143	
≥ 28 u/ml	2	0	0	2	

* statistically significant; N/A, not applicable.

### Clinicopathological relevance of sIL-8 levels in gastric cancer

**Figure 1:**
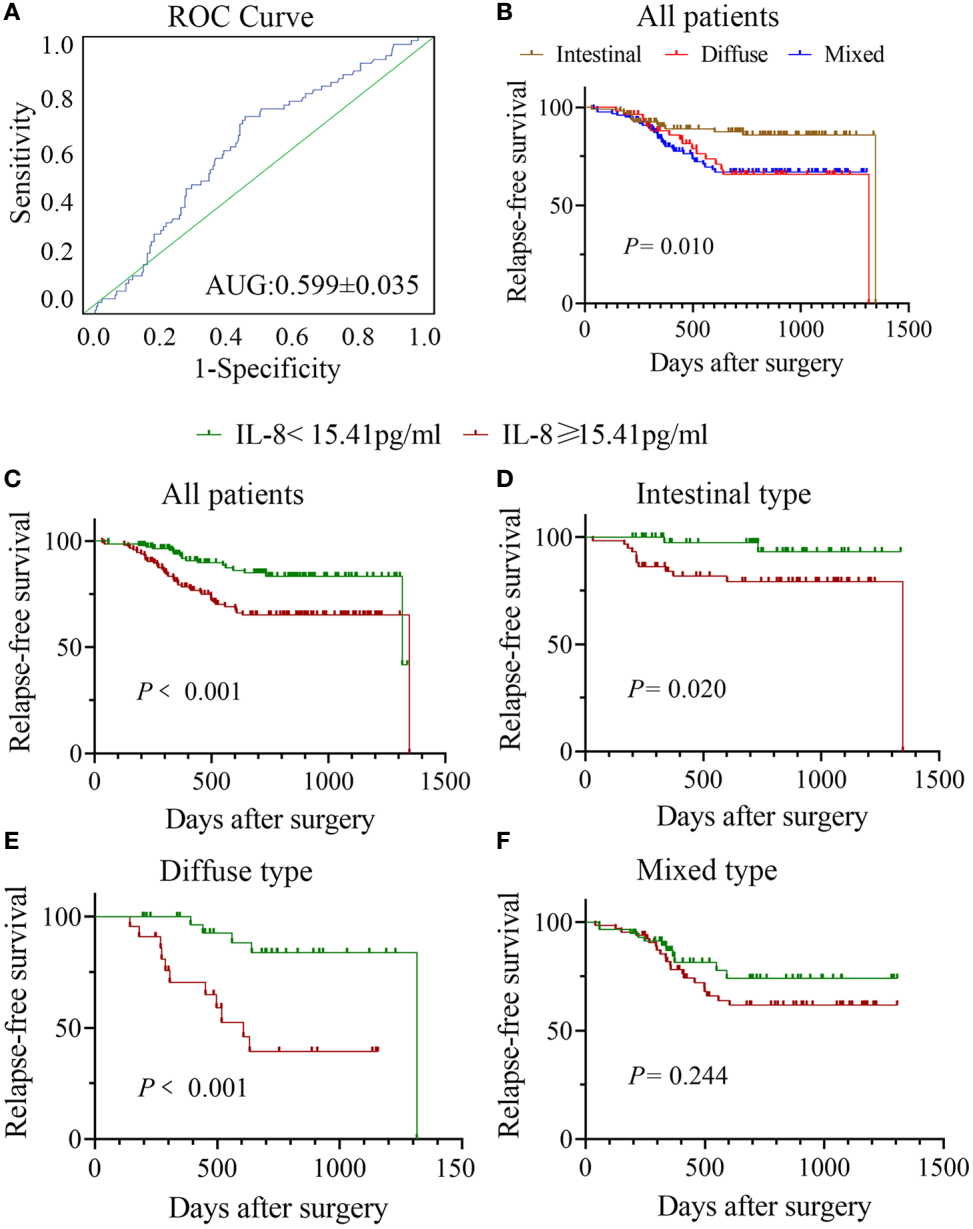
Correlation between sIL-8 and RFS in gastric cancer. **(A)** ROC analysis indicated that the sIL-8 value less than 15.41 pg/ml was defined as low otherwise as high. **(B)** Patients with intestinal-type tumor had much better RFS than those with diffuse- and mixed-type diseases (*P* = 0.010), who showed no differences in RFS. **(C)** Patients with high sIL-8 level had worse RFS than those with low sIL-8 level in the whole cohort (*P* < 0.001). **(D)** The sIL-8 levels had survival significance in patients with intestinal-type tumor (*P* = 0.020). **(E)** Patients with high sIL-8 level had worse RFS than those with low sIL-8 level in the diffuse-type tumor (*P* < 0.001). **(F)** In the mixed-type tumor, sIL-8 levels had no survival significance (*P* = 0.244).

Previously, we demonstrated that high serum IL-8 level in gastric cancer patients is associated with poor response to chemotherapy (4). To elucidate the clinicopathological significance of sIL-8 in gastric cancer, sIL-8 levels of these enrolled patients were measured using ELISA. The sIL-8 levels ranged from 1.48 pg/ml to 1025.22 pg/ml. According to the ROC analysis, a sIL-8 value less than 15.41 pg/ml was defined as low otherwise as high (**Figure 1A**). High sIL-8 level was strongly associated with Lauren classification (*P* = 0.029). Importantly, patients with high sIL-8 levels were more predisposed to tumor relapse (*P* < 0.001) (**Table 2**). High sIL-8 levels correlated with LNM, but not with remarkable significance (*P* = 0.052). No significant correlations of sIL-8 levels with the classic tumor markers, such as CEA, AFP, CA199, CA125 and CA153, were observed (*P* > 0.05).

**Table 2:** Clinicopathological relevance of serum interleukin-8 (sIL-8) level in gastric cancer.

Clinicopathological features	N	All patients		*P*	N	Intestinal		*P*	N	Diffuse		*P*	N	Mixed		P
		sIL-8< 15.41 (pg/ml)	sIL-8≥ 15.41 (pg/ml)			sIL-8< 15.41 (pg/ml)	sIL-8≥ 15.41 (pg/ml)			sIL-8 < 15.41 (pg/ml)	sIL-8 ≥ 15.41 (pg/ml)			sIL-8< 15.41 (pg/ml)	sIL-8≥ 15.41 (pg/ml)	
Age (y)				0.142				0.832				0.388				0.420
≤60	105	57	48		33	14	19		37	25	12		35	18	17	
> 60	230	105	125		92	41	51		28	16	12		110	48	62	
Sex				0.703				0.714				0.457				0.806
male	239	114	125		104	45	59		31	21	10		104	48	56	
female	96	48	48		21	10	11		34	20	14		41	18	23	
Depth of invasion (T)				0.177				0.057				0.005*				0.294
T1	90	49	41		54	29	25		11	11	0		25	9	16	
T2–T4	245	113	132		71	26	45		54	30	24		120	57	63	
LNM				0.052				0.008*				< 0.001*				0.158
N0	137	75	62		77	41	36		21	20	1		39	14	25	
N1–N3	198	87	111		48	14	34		44	21	23		106	52	54	
Distant metastasis (M)				N/A				N/A				N/A				N/A
M0	335	162	173		125	55	70		65	41	24		145	66	79	
M1	0	0	0		0	0	0		0	0	0			0	0	
TNM stage				0.218				0.123				0.005*				0.685
I	107	57	50		63	32	31		11	11	0		33	14	19	
II–IV	228	105	123		62	23	39		54	30	24		112	52	60	
Lauren classification				0.029*	/				/				/			
intestinal	125	55	70													
diffuse	65	41	24													
mixed	145	66	79													
Recurrence				< 0.001*				0.007*				0.005*				0.045*
recurrence-free	262	141	121		109	53	56		46	34	12		107	54	53	
recurrence	73	21	52		16	2	14		19	7	12		38	12	26	
CEA				0.577				0.280				1.000				0.048*
< 5 ng/ml	291	139	152		104	48	56		58	36	22		129	55	74	
≥ 5 ng/ml	44	23	21		21	7	14		7	5	2		16	11	5	
AFP				0.724				1.000				1.000				0.689
< 25 μg/l	327	159	168		124	55	69		64	40	24		139	64	75	
≥ 25 μg/l	8	3	5		1	0	1		1	1	0		6	2	4	
CA125				0.676				0.404				N/A				1.000
< 35 u/ml	324	156	168		119	51	68		65	41	24		140	64	76	
≥ 35 u/ml	11	6	5		6	4	2		0	0	0		5	2	3	
CA199				0.602				1.000				0.662				0.702
< 37 u/ml	301	147	154		114	50	64		59	38	21		128	59	69	
≥ 37 u/ml	34	15	19		11	5	6		6	3	3		17	7	10	
CA153				1.000				N/A				N/A				1.000
< 28 u/ml	333	161	172		125	55	70		65	41	24		143	65	78	
≥ 28 u/ml	2	1	1		0	0	0		0	0	0		2	1	1	

* statistically significant; N/A, not applicable.

To precisely investigate the clinical relevance of sIL-8 levels, stratified analyses were performed according to Lauren classification. As shown in **Table 2**, high sIL-8 levels correlated with LNM in the intestinal- (*P* = 0.008) and diffuse- (*P* < 0.001) type tumors; however, this correlation was not observed in the mixed-type disease (*P* = 0.158). Its relevance with tumor recurrence persisted in the intestinal-, diffuse- and mixed-type tumors, indicating a close positive association of sIL-8 with tumor recurrence. Importantly, the negative association was observed between sIL-8 level and serum CEA in the mixed-type tumor (*P* = 0.048), suggesting that sIL-8 is complementary to CEA as a tumor marker. Furthermore, high sIL-8 levels correlated with advanced T stage (*P* = 0.005) and TNM stage (*P* = 0.005) in the diffuse-type tumor rather than in the intestinal- or mixed-type tumors.

### sIL-8 level as an independent risk factor for LNM in gastric cancer

To clarify the role of sIL-8 in gastric cancer LNM, risk factors for LNM were analyzed. Univariate analyses in the whole cohort indicated that older age (*P* = 0.001), T stage (*P* < 0.001), Lauren classification (*P* < 0.001), CEA (*P* = 0.009), and CA199 (*P* = 0.011) were clinicopathological factors associated with LNM (**Table 3**), whereas the significance of sIL-8 was not remarkable (*P* = 0.052). Multivariate analyses showed that T stage (*P* < 0.001), Lauren classification (*P* = 0.005), and CEA (*P* = 0.043) were independent risk factors for LNM (**Table 4**).

**Table 3:** Associated clinicopathological factors for LNM in gastric cancer.

Clinicopathological features	N	All patients		*P*	N	Intestinal		*P*	N	Diffuse		*P*	N	Mixed		*P*
		N0	N1-N3			N0	N1-N3			N0	N1-N3			N0	N1-N3	
Age (y)				0.001*				0.001*				0.030*				0.117
≤ 60	105	57	48		33	28	5		37	16	21		35	13	22	
> 60	230	80	150		92	49	43		28	5	23		110	26	84	
Sex				0.501				0.013*				0.993				0.412
male	239	95	144		104	59	45		31	10	21		104	26	78	
female	96	42	54		21	18	3		34	11	23		41	13	28	
Depth of invasion (T)				< 0.001*				< 0.001*				< 0.001*				< 0.001*
T1	90	77	13		54	50	4		11	10	1		25	17	8	
T2–T4	245	60	185		71	27	44		54	11	43		120	22	98	
Lauren classification				< 0.001*	/				/				/			
intestinal	125	77	48													
diffuse	65	21	44													
mixed	145	39	106													
IL-8				0.052				0.008*				< 0.001*				0.158
< 15.41 pg/ml	162	75	87		55	41	14		41	20	21		66	14	52	
≥ 15.41 pg/ml	173	62	111		70	36	34		24	1	23		79	25	54	
CEA				0.009*				0.015*				0.413				0.070
< 5 ng/ml	291	127	164		104	69	35		58	20	38		129	438	91	
≥ 5 ng/ml	44	10	34		21	8	13		7	1	6		16	1	15	
AFP				0.148				0.384				0.323				0.192
< 25 μg/l	327	136	191		124	77	47		64	20	44		139	39	100	
≥ 25 μg/l	8	1	7		1	0	1		1	1	0		6	0	6	
CA125				0.535				0.031*				N/A				0.611
< 35 u/ml	324	134	190		119	76	43		65	21	44		140	37	103	
≥ 35 u/ml	11	3	8		6	1	5		0	0	0		5	2	3	
CA199				0.011*				0.332				0.166				0.158
< 37 u/ml	301	130	171		114	72	42		59	21	38		128	37	91	
≥ 37 u/ml	34	7	27		11	5	6		6	0	6		17	2	15	
CA153				0.515				N/A				N/A				1.000
< 28 u/ml	333	137	196		125	77	48		65	21	44		143	39	104	
≥ 28 u/ml	2	0	2		0	0	0		0	0	0		2	0	2	

* statistically significant; N/A, not applicable.

**Table 4:** Univariate and multivariate analyses of risk factors for LNM in gastric cancer according to Lauren classification.

Clinicopathological features	Univariate analysis		Multivariate analysis	
	OR (95% CI)	*P*	OR (95% CI)	*P*
All patients				
Age (> 60y)	2.227 (1.391-3.563)	0.001*	1.663 (0.875-3.160)	0.120
Sex (female)	0.848 (0.525-1.370)	0.501	–	–
T (T2–T4)	18.263 (9.478-35.189)	<0.001*	12.838 (6.410-25.712)	< 0.001*
Lauren classification				
intestinal	1.000 (Ref)		1.000 (Ref)	
diffuse	3.361 (1.785-6.327)	<0.001*	3.152 (1.410-7.046)	0.005*
mixed	4.360 (2.607-7.293)	<0.001*	3.786 (2.028-7.067)	< 0.001*
IL-8 (≥ 15.41 pg/ml)	1.543 (0.996-2.392)	0.052	–	–
CEA (≥ 5 ng/ml)	2.633 (1.253-5.530)	0.011*	2.624 (1.029-6.687)	0.043*
CA199 (≥ 37 u/ml)	2.932 (1.238-6.944)	0.014*	1.113 (0.436-2.840)	0.823
Intestinal type				
Age (> 60y)	4.914 (1.744-13.848)	0.003*	1.493 (0.367-6.069)	0.576
Sex (female)	0.219 (0.061-0.788)	0.020*	0.159 (0.038-0.665)	0.012*
T (T2–T4)	20.370 (6.610-62.777)	<0.001*	18.619 (5.001-69.325)	< 0.001*
IL-8 (≥ 15.41 pg/ml)	2.766 (1.285-5.954)	0.009*	2.769 (1.040-7.374)	0.042*
CEA (≥ 5 ng/ml)	3.204 (1.214-8.452)	0.019*	2.381 (0.712-7.955)	0.159
CA199 (≥ 37 u/ml)	2.057 (0.592-7.154)	0.257	–	–
Diffuse type				
Age (> 60y)	3.505 (1.093-11.241)	0.035*	2.716 (0.650-11.352)	0.171
Sex (female)	0.996 (0.352-2.819)	0.993	–	–
T (T2–T4)	39.091 (4.510-338.850)	0.001*	16.805 (1.828-154.455)	0.013*
IL-8 (≥ 15.41 pg/ml)	21.905 (2.699-177.750)	0.004*	11.907 (1.372-103.326)	0.025*
CEA (≥ 5 ng/ml)	3.158 (0.355-28.080)	0.302	–	–
CA199 (≥ 37 u/ml)		0.999	–	–
Mixed type				
Age (> 60y)	1.909 (0.845-4.311)	0.120	–	–
Sex (female)	0.718 (0.325-1.588)	0.413	–	–
T (T2–T4)	9.466 (3.628-24.701)	<0.001*	9.466 (3.628-24.701)	< 0.001*
IL-8 (≥ 15.41 pg/ml)	0.582 (0.273-1.240)	0.160	–	–
CEA (≥ 5 ng/ml)	6.264 (0.799-49.112)	0.081	–	–
CA199 (≥ 37 u/ml)	3.049 (0.664-14.000)	0.125	–	–

* statistically significant.

Further stratified analyses showed that older age (*P* = 0.003), female sex (*P* = 0.020), T stage (*P* < 0.001), sIL-8 (*P* = 0.009), and CEA (*P* = 0.019) were clinicopathological factors in the patients with intestinal-type tumor (**Table 3**) and female sex (*P* = 0.012), T stage (*P* < 0.001) and sIL-8 (*P* = 0.042) were independent risk factors (**Table 4**). In the patients with diffuse-type tumor, older age (*P* = 0.035), T stage (*P* = 0.001), and sIL-8 (*P* = 0.004) were factors (**Table 3**), but only T stage (*P* = 0.013) and sIL-8 (*P* = 0.025) were independent risk factors (**Table 4**). In the patients with mixed-type tumor, only T stage was a risk factor for LNM (*P* < 0.001) (**Tables 3** and **4**). CEA was not an independent risk factor for LNM in any type (*P* > 0.05) although it acted as an independent factor in the whole cohort. However, sIL-8 was an independent risk factor in patients with intestinal- and diffuse-type tumors although it was not in the whole patients.

### High sIL-8 level was an independent risk factor for tumor recurrence in gastric cancer

To confirm the role of sIL-8 in tumor recurrence of gastric cancer, risk factors for tumor relapse were analyzed. Among all these enrolled patients, tumor recurrence occurred in 73 patients (21.79%) during follow-up. RFS analyses showed that patients with intestinal-type tumor had much better RFS than those with diffuse- and mixed-type diseases (*P* = 0.010), and that patients with diffuse- and mixed-type tumors had similar worse RFS (**Figure 1B**). Patients with high sIL-8 levels had worse RFS than those with low sIL-8 levels (*P* < 0.001, **Figure 1C**). Stratified analyses according to Lauren classification showed that sIL-8 levels had survival significance in the intestinal- (*P* = 0.020, **Figure 1D**) and diffuse-type tumors (*P* < 0.001, **Figure 1E**) but not in mixed-type tumor (*P* = 0.244, **Figure 1F**).

As shown in **Table 5**, older patients were prone to tumor relapse (*P* = 0.005). Advanced T stage (*P* < 0.001), N stage (*P* < 0.001), TNM stage (*P* < 0.001), and diffuse-type diseases (*P* = 0.008) preluded tumor recurrence. sIL-8 (*P* < 0.001), CEA (*P* = 0.001), and CA199 (*P* = 0.044) levels were positively associated with tumor recurrence. Multivariate analyses showed that Lauren classification (*P* = 0.010), sIL-8 (*P* < 0.001), and CEA (*P* = 0.003) were independent risk factors for tumor recurrence (**Table 6**).

**Table 5:** Clinicopathological factors associated with tumor recurrence in gastric cancer.

Clinicopathological features	N	All patients		*P*	N	Intestinal		*P*	N	Diffuse		*P*	N	Mixed		*P*
		R-free	R			R-free	R			R-free	R			R-free	R	
Age (y)				0.005*				0.067				0.317				0.006*
≤ 60	105	92	13		33	32	1		37	28	9		35	32	3	
>60	230	170	60		92	77	15		28	18	10		110	75	35	
Sex				0.574				0.305				0.260				0.047*
male	239	185	54		104	89	15		31	24	7		104	72	32	
female	96	77	19		21	20	1		34	22	12		41	35	6	
Depth of invasion (T)				<0.001*				0.001*				0.486				0.006*
T1	90	86	4		54	53	1		11	9	2		25	24	1	
T2–T4	245	176	69		71	56	15		54	37	17		120	83	37	
Lymph node metastasis				<0.001*				<0.001*				0.016*				0.002*
N0	137	130	7		77	75	2		21	19	2		39	36	3	
N1–N3	198	132	66		48	34	14		44	27	17		106	71	35	
Distant metastasis (M)				N/A				N/A				N/A				N/A
M0	335	262	73		125	109	16		65	46	19		145	107	38	
M1	0	0	0		0	0	0		0	0	0		0	0	0	
TNM stage				<0.001*				<0.001*				0.154				0.001*
I	107	104	3		63	62	1		11	10	1		33	32	1	
II–IV	228	158	70		62	47	15		54	36	18		112	75	37	
Lauren classification				0.008*	/				/				/			
intestinal	125	109	16					
diffuse	65	46	19					
mixed	145	107	38					
IL-8				<0.001*				0.007*				0.005*				0.045*
< 15.41 pg/ml	162	141	21		55	53	2		41	34	7		66	54	12	
≥ 15.41 pg/ml	173	121	52		70	56	14		24	12	12		79	53	26	
CEA				0.001*				0.029*				0.663				< 0.001*
< 5 ng/ml	291	237	54		104	94	10		58	40	18		129	103	26	
≥ 5 ng/ml	44	25	19		21	15	6		7	6	1		16	4	12	
AFP				0.072				1.000				1.000				0.041*
< 25 μg/l	327	258	69		124	108	16		64	45	19		139	105	34	
≥ 25 μg/l	8	4	4		1	1	0		1	1	0		6	2	4	
CA125				0.710				0.170				N/A				1.000
< 35 u/ml	324	254	70		119	105	14		65	46	19		140	103	37	
≥ 35 u/ml	11	8	3		6	4	2		0	0	0		5	4	1	
CA199				0.044*				0.632				0.347				0.149
< 37 u/ml	301	240	61		114	100	14		59	43	16		128	97	31	
≥ 37 u/ml	34	22	12		11	9	2		6	3	3		17	10	7	
CA153				0.047*				N/A				N/A				0.067
< 28 u/ml	333	262	71		125	109	16		65	46	19		143	107	36	
≥ 28 u/ml	2	0	2		0	0	0		0	0	0		2	0	2	

R, recurrence; R-free, recurrence-free. * statistically significant; N/A, not applicable.

**Table 6:** Univariate and multivariate analyses of risk factors for tumor recurrence in gastric cancer according to Lauren classification.

Clinicopathological features	Univariate analysis		Multivariate analysis	
	OR (95% CI)	*P*	OR (95% CI)	*P*
All patients				
Age (> 60y)	2.498 (1.303-4.790)	0.006*	1.873 (0.857-4.094)	0.116
Sex (female)	0.845 (0.470-1.520)	0.575	–	–
T (T2–T4)	8.429 (2.978-23.858)	<0.001*	1.416 (0.357-5.614)	0.620
N (N1–N3)	9.286 (4.107-20.996)	<0.001*	2.579 (0.903-7.365)	0.077
TNM (II–IV)	15.359 (4.711-50.072)	<0.001*	3.656 (0.657-20.342)	0.139
Lauren classification				
intestinal	1.000 (Ref)		1.000 (Ref)	
diffuse	2.814 (1.330-5.951)	0.007*	3.440 (1.335-8.859)	0.010*
mixed	2.419 (1.273-4.598)	0.007*	2.113 (0.990-4.512)	0.053
IL-8 (≥ 15.41 pg/ml)	2.885 (1.645-5.061)	<0.001*	3.558 (1.855-6.823)	<0.001*
CEA (≥ 5 ng/ml)	3.336 (1.714-6.491)	<0.001*	3.483 (1.545-7.852)	0.003*
CA199 (≥ 37 u/ml)	2.146 (1.006-4.577)	0.048*	1.133 (0.489-2.629)	0.771
Intestinal type				
Age (> 60y)	6.234 (0.790-49.194)	0.083	–	–
Sex (female)	0.297 (0.037-2.378)	0.253	–	–
T (T2–T4)	14.196 (1.812-111.251)	0.012*	0.919 (0.011-76.096)	0.970
N (N1–N3)	15.441 (3.324-71.740)	<0.001*	4.059 (0.669-24.611)	0.128
TNM (II–IV)	19.787 (2.523-155.161)	0.004*	7.316 (0.074-721.742)	0.396
IL-8 (≥ 15.41 pg/ml)	6.625 (1.437-30.548)	0.015*	5.458 (1.041-28.615)	0.045*
CEA (≥ 5 ng/ml)	3.760 (1.191-11.869)	0.024*	2.594 (0.691-9.729)	0.158
CA199 (≥ 37 u/ml)	1.587 (0.311-8.110)	0.579	–	–
Diffuse type				
Age (> 60y)	1.728 (0.588-5.078)	0.320	–	–
Sex (female)	1.870 (0.624-5.602)	0.263	–	–
T (T2–T4)	2.068 (0.403-10.619)	0.384	–	–
N (N1–N3)	5.981 (1.234-28.992)	0.026*	3.428 (0.621-18.925)	0.158
TNM (II–IV)	5.000 (0.593-42.162)	0.139	–	–
IL-8 (≥ 15.41 pg/ml)	4.857 (1.552-15.203)	0.007*	3.155 (0.912-10.911)	0.070
CEA (≥ 5 ng/ml)	0.370 (0.041-3.306)	0.347	–	–
CA199 (≥ 37 u/ml)	2.687 (0.491-14.713)	0.254	–	–
Mixed type				
Age (> 60y)	4.978 (1.427-17.367)	0.012*	2.861 (0.742-11.024)	0.127
Sex (female)	0.386 (0.148-1.008)	0.052	–	–
T (T2–T4)	10.699 (1.395-82.079)	0.023*	3.925 (0.437-35.259)	0.222
N (N1–N3)	5.915 (1.703-20.553)	0.005*	1.527 (0.271-8.614)	0.631
TNM (II–IV)	15.787 (2.076-120.072)	0.008*	6.414 (0.455-90.329)	0.168
IL-8 (≥ 15.41 pg/ml)	2.208 (1.010-4.825)	0.047*	4.781 (1.633-13.996)	0.004*
CEA (≥ 5 ng/ml)	11.885 (3.542-39.878)	<0.001*	14.924 (3.330-66.887)	<0.001*
CA199 (≥ 37 u/ml)	2.190 (0.769-6.241)	0.142	–	–

* statistically significant.

Stratified analyses showed that the connection of older patients with tumor recurrence only occurred in the mixed-type tumor (*P* = 0.006), and male patients were more likely to suffer recurrence (*P* = 0.047). T and TNM stages had not significantly associated with relapse in patients with diffuse-type tumor, whereas N stage correlated to tumor relapse in all types. The sIL-8 level was also positively associated with tumor recurrence in all types. CEA lost its significant connection with tumor relapse in the diffuse-type tumor, and CA199 did not predict tumor recurrence in these three types respectively. However, AFP obtained positive association in the mixed-type tumor (*P* = 0.041) (**Table 5**). Multivariate analyses indicated that only sIL-8 was an independent risk factor for tumor recurrence in the intestinal-type tumor, that no independent risk factor was observed in the diffuse-type tumor, and that sIL-8 (*P* = 0.004) and CEA (*P* < 0.001) were independent risk factors for tumor relapse in the mixed-type tumor (**Table 6**).

### sIL-8 acting as a potential marker for gastric cancer with negative CEA

CEA is one of the prominent classic tumor markers for gastrointestinal tumors, and is commonly used in screening, predicting the prognosis, and monitoring gastric cancer. CEA has been reported to be increased in approximately 16% – 68% in gastric cancer patients (21). In our cohort, increased CEA level was detected in 13.13% of enrolled patients. As shown in **Table 7**, in patients with negative CEA, sIL-8 showed remarkable significance for LNM or tumor recurrence in all patients or in subtypes except in mixed-type for LNM. However, sIL-8 had no predictive value for LNM or tumor recurrence in patients with increased CEA level. These results indicated that sIL-8 may be a useful tumor markers candidate in gastric cancer patients with negative CEA.

**Table 7:** Relevance of combing IL-8 and CEA with tumor recurrence or LNM in gastric cancer.

Lauren classification	CEA (ng/ml)		< 5		*P*	> 5		*P*
	IL-8 (pg/ml)		< 15.41	≥ 15.41		< 15.41	≥ 15.41	
All patients	LNM	N0	70	57	0.033*	5	5	>0.999
		N1–N3	69	95		18	16	
	recurrence	–	128	109	<0.001*	13	12	0.967
		+	11	43		10	9	
Intestinal type	LNM	N0	38	31	0.013*	3	5	>0.999
		N1–N3	10	25		4	9	
	recurrence	–	47	47	0.019*	6	9	0.613
		+	1	9		1	5	
Diffuse type	LNM	N0	19	1	<0.001*	1	0	>0.999
		N1–N3	17	21		4	2	
	recurrence	–	30	10	0.004*	4	2	>0.999
		+	6	12		1	0	
Mixed type	LNM	N0	13	25	0.245	1	0	>0.999
		N1–N3	42	49		10	5	
	recurrence	–	51	52	0.002*	3	1	>0.999
		+	22	4	8	4

LNM, lymph node metastasis. * statistically significant.

## Discussion

In this study, comprehensive analysis of clinicopathological data of 335 patients with gastric cancer was performed, predominantly in the profile of sIL-8 level and its clinicopathological relevance. To the best of our knowledge, our study is the first to suggest that the sIL-8 level acts as a potential predictor for LNM and tumor recurrence for gastric cancer, especially in patients with negative CEA, although further prospective studies are warranted.

Several studies have evaluated the probability of serum IL-8 as a prognostic marker of different cancer types. In a study of 68 patients with pancreatic cancer, high serum IL-8 level was found to be strongly associated with poor prognosis and can be regarded as a useful tumor marker (23). A phase II clinical trial, monitoring the sIL-8 levels of 58 patients with metastatic breast cancer before and during the first-line chemotherapy, indicated that patients with lower sIL-8 level (< 16.6 pg/ml) had a significantly higher rate of overall survival than those with higher sIL-8 level (24). Schalper et al. measured the baseline sIL-8 levels in samples from 1344 patients with advanced renal cell carcinoma, melanoma or non-small cell lung cancer treated with nivolumab and/or ipilimumab, everolimus or docetaxel from four phase III clinical trials, showing that elevated baseline sIL-8 levels (≥ 23 pg/ml) are associated with poor outcomes in patients across all tumor types (25).

Herein, we revealed that the sIL-8 level is strongly associated with Lauren classification in gastric cancer, and patients with intestinal-type tumor have higher sIL-8 level than those with diffuse-type tumor. Intestinal-type gastric cancer originates primarily from atrophic gastritis and/or intestinal metaplasia caused mainly by *Helicobacter Pylori* (*H. pylori*)-induced chronic inflammation (26, 27). *H. pylori* infection can lead to increased serum IL-8 levels (28, 29). Thus, high sIL-8 level mainly occurs in the intestinal-type tumor as expected. However, the underlying detailed molecular mechanisms remain to be investigated. Increased sIL-8 levels in gastric cancer patients has been reported to originate from tumor stromal cells, including mesenchymal stem cells (MSCs) (30) or TAMs (10), and some reports showed that sIL-8 may be produced by tumor cells (31). Our previous studies indicated that sIL-8 in gastric cancer patients is prominently produced by CAFs in tumor tissues, that CAFs secreted more IL-8 than the normal fibroblasts, and that sIL-8 usually returns to a normal level within one month after radical gastrectomy (4). Given that the proportion of CAFs is much higher than that of MSCs, TAMs and other myeloid cells in the gastric cancer tumor microenvironment (TME), the sIL-8 mainly originates from CAFs. We are conducting further studies to investigate how tumor cells upregulate the IL-8 expression of CAFs in the gastric cancer TME.

Our studies have clearly demonstrated that a higher sIL-8 level is an independent risk factor of LNM or tumor relapse in gastric cancer, both in the intestinal- and diffuse-type tumors, suggesting that the increased IL-8 level plays profound roles in tumor progression irrespective of tumor classification. IL-8 has been shown to induce PD-L1 expression in gastric cancer cells *via* c-Myc regulated by STAT3/mTOR signaling activation, resulting in immune escape of tumor cells (30). Furthermore, IL-8 can induce PD-L1 expression in macrophages, which contributes to the immunosuppressive microenvironment in gastric cancer (10). Our recent studies revealed that IL-8 promotes LNM *via* PD-1 upregulation in CD8^+^ T cells (32). Furthermore, IL-8 can enhance the metastatic capacity of colorectal cancer cells by inducing epithelial-to-mesenchymal transition through the IL-8/p65 signaling pathway (33). Our current research also indicated that more neutrophils were accumulated in gastric cancer TME due to chemotaxis of increased IL-8, and these tumor-associated neutrophils promote tumor LNM *via* mediating EMT of tumor cells (unpublished data). Taken together, increased IL-8 level can promote tumor progression by inducing tumor escape or immune tolerance, and enhancing tumor invasiveness, in addition to inducing chemoresistance, which results in poor prognosis of gastric cancer patients.

Hence, tumor-derived IL-8 has been considered as a potential therapeutic target for cancer treatment (34). Transcriptional repression of IL-8 promoter activity using DACH1 or treatment with IL-8 antagonists can provide a favorable survival for lung cancer patients (35). IL-8 primarily acts through its receptors, CXCR1 and CXCR2 (5, 11). CXCR2 signaling has been shown to be an excellent therapeutic target for pancreatic cancer (36). Suppressing IL-8 or blocking the IL-8/CXCR2 axis with IFN-γ can enhance the anti-PD-1 efficacy in pancreatic cancer (37). Thus, targeting tumor-derived IL-8 may be a novel strategy to improve therapeutic outcomes of gastric cancer, although further research is needed.

In addition, the relation of IL-8 with the classic tumor biomarkers, including CEA, AFP, CA199, CA125 and CA153, was evaluated. The sIL-8 level was not positively associated with these classic markers. Among these markers, only CEA showed clinical significance with LNM or tumor relapse. High sIL-8 level was an independent risk factor for LNM in both intestinal- and diffuse-type tumors but not in the whole cohort, whereas CEA was not an independent risk factor for LNM in any type tumor although it acted as one of the independent risk factors in the whole cohort. Patients with a high sIL-8 level had worse RFS than those with low sIL-8 level in both intestinal- and diffuse-types, and sIL-8 was also an independent risk factor for recurrence in the intestinal-type. Both sIL-8 and CEA were independent risk factors for tumor relapse in the mixed-type tumor. Importantly, we found that sIL-8 has no predictive value for LNM or tumor recurrence in patients with high CEA level, but shows positive association with LNM or tumor recurrence in patients with negative CEA. These results suggested that sIL-8 is a promising tumor biomarker for gastric cancer patients with negative CEA, which effectively complements the lack of markers in CEA-negative patients.

Although this is only a clinical observation study, it examined a relatively large patient volume. We aim to perform a related clinical trial to ascertain the role of sIL-8 as a tumor marker for gastric cancer. Elevated serum CEA level is definitely associated with the existence of cancerous diseases; however, several conditions other than cancers may cause elevation of sIL-8 level. Thus, an increased sIL-8 level does not necessarily indicate tumor occurrence. However, if gastric cancer is already present, high sIL-8 levels indicate that patients are more prone to LNM and poor prognosis.

In summary, the present study demonstrated the predictive role of sIL-8 in LNM and tumor recurrence of gastric cancer. Given its important role in gastric cancer progression, novel strategies targeting tumor-derived IL-8 may achieve promising therapeutic effects for gastric cancer.

## Data availability statement

The original contributions presented in the study are included in the article/Supplementary Material. Further inquiries can be directed to the corresponding author.

## Ethics statement

The studies involving human participants were reviewed and approved by the Institutional Review Board of Nanjing University of Chinese Medicine. The patients/participants provided their written informed consent to participate in this study.

## Author contributions

LS and JZ conceived the study. XL, GX, JZ and YH developed methodology. XL, GX, JZ and YW performed the experiments. LS, XL, GX and JZ analyzed the data. LS and XL wrote the manuscript. LS provided financial support. All authors read and approved the final manuscript. All authors contributed to the article and approved the submitted version.

## Funding

This work was supported by the National Natural Science Foundation of China (81871959), the Key R & D Program of Jiangsu Province (Social Development, BE2018758), the Key Medical Talents Program of Jiangsu Province (ZDRCA2016014) and the Programs of Jiangsu Province Hospital of Chinese Medicine (Y2018RC14) and Jiangsu Innovation Program for Postgraduate (KYCX22_1925).

## Conflict of interest

The authors declare that the research was conducted in the absence of any commercial or financial relationships that could be construed as a potential conflict of interest.

## Publisher’s note

All claims expressed in this article are solely those of the authors and do not necessarily represent those of their affiliated organizations, or those of the publisher, the editors and the reviewers. Any product that may be evaluated in this article, or claim that may be made by its manufacturer, is not guaranteed or endorsed by the publisher.
